# Urinary extracellular vesicles. A promising shortcut to novel biomarker discoveries

**DOI:** 10.1007/s00441-017-2621-0

**Published:** 2017-04-20

**Authors:** Karina Barreiro, Harry Holthofer

**Affiliations:** 1 Institute for Molecular Medicine Finland (FIMM), University of Helsinki, Helsinki, Finland; 2 Freiburg Institute for Advanced Studies, Albert-Ludwigs University Freiburg, Freiburg, Germany

**Keywords:** Kidney disease, Biomarkers, MicroRNA, Extracellular vesicles, Urine

## Abstract

Proteomic and genomic techniques have reached full maturity and are providing unforeseen details for the comprehensive understanding of disease pathologies at a fraction of previous costs. However, for kidney diseases, many gaps in such information remain to inhibit major advances in the prevention, treatment and diagnostics of these devastating diseases, which have enormous global impact. The discovery of ubiquitous extracellular vesicles (EV) in all bodily fluids is rapidly increasing the fundamental knowledge of disease mechanisms and the ways in which cells communicate with distant locations in processes of cancer spread, immunological regulation, barrier functions and general modulation of cellular activity. In this review, we describe some of the most prominent research streams and findings utilizing urinary extracellular vesicles as highly versatile and dynamic tools with their extraordinary protein and small regulatory RNA species. While being a highly promising approach, the relatively young field of EV research suffers from a lack of adherence to strict standardization and carefully scrutinized methods for obtaining fully reproducible results. With the appropriate guidelines and standardization achieved, urine is foreseen as forming a unique, robust and easy route for determining accurate and personalized disease signatures and as providing highly useful early biomarkers of the disease pathology of the kidney and beyond.

## Introduction

Following extensive genome-wide analyses and the full maturation of integrative technologies such as RNA-sequencing (RNA-Seq) techniques to reveal the crucial regulatory functions of the “non-coding” DNA bulk of human and other species (Lee et al. 2010), recent years have seen strong advances in a variety of organ functions with unprecedented molecular accuracy. Nevertheless, an extensive understanding of, for example, key kidney functions, including mechanisms of glomerular filtration and the formation of urine from primary urine to the final voided urine, remains to be achieved (Saritas et al. 2015; Maas et al. 2016). The combined use of proteomics, lipidomics, metabolomics and comprehensive genomic analyses, together with advanced systems biology algorithms, offers the promise of major new discoveries for the benefit of patients with kidney disease.

Urine formation provides a key route for waste removal from the body. In addition to immediate metabolic waste products, variable amount of electrolytes, small peptides and larger functional proteins (Hildonen et al. 2016) are removed. Based, for example, on hydration status, diet and excercise, the electrolyte concentration may variate considerably. For years, this dynamic nature of urine has presented a challenge for the full exploitation of the potential of this bodily fluid, which is easy to collect.

The structural basis of glomerular filtration has been defined down to the fine molecular level, including the identification of the respective signaling and functional pathways, whereas the functional mechanisms remain to be fully understood (Patrakka and Tryggvason 2010; Pollak et al. 2014; Scott and Quaggin 2015). At the same time, however, the continuous global increase of chronic kidney diseases (CKD) attributable to a variety of causes involves over 10 % of most populations and geographical areas (El Nahas 2005; Remuzzi et al. 2006). These alarming numbers call for practical advances leading to early diagnostics, better therapies and cost-efficient personalized disease management even in less fortunate areas and countries. Many of the technologies required are beginning to reach their full maturity for the achievement of these goals.

The increase of CKD is mostly the result of the rapid increase of diabetes world-wide (El Nahas 2005). However, patient subpopulations susceptible to life-threatening diabetic end-organ damage mostly cannot be identified early enough to prevent the progress of disease. Apart from the medicinal modulation of the renin-angiotensin axis (Remuzzi et al. 2006), only a few other CKD disease mechanisms have been identified and, accordingly, the repertoire of targeted medication available to halt disease progression is not satisfactory. Currently, the exhaustive genetic data produced have provided few clues to the factors and mechanisms for the identification of the vulnerable target subpopulations or even of those who might benefit from the currently available treatment options. In spite of numerous proposed early biomarkers to monitor disease progression and activity, none have been fully validated for wide clinical use.

The discovery of vesicles of various sizes secreted from practically all cell types in the body (Thery et al. 2009; Yanez-Mo et al. 2015) is rapidly revolutionizing our understanding of key biological phenomena including organ growth and development, cancer and metastasis, cellular homeostasis, immunological defense, barrier functions to the exterior and, importantly, intercellular communication (for references, see Thery et al. 2009, van der Pol et al. 2012). Although this is a relatively young field, many new cellular-intercellular mechanisms including the spread of viral infections (Nour and Modis 2014) and the spread of cancer cells (Tomasetti et al. 2017) can be explained by messages from extracellular vesicles (EV).

## Extracellular vesicles

**Fig. 1:**
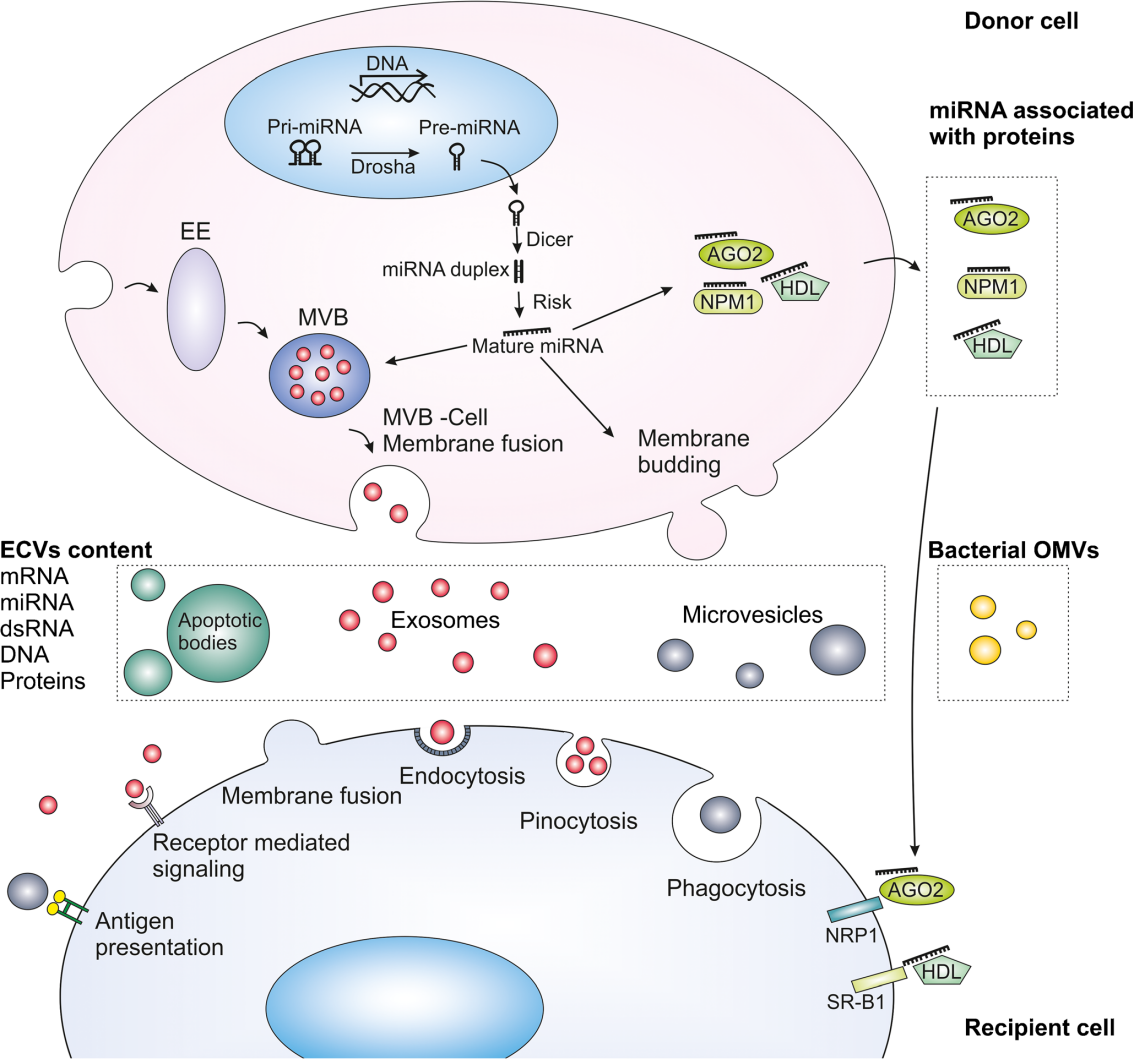
Biogenesis of microRNA (*miRNA*), extracellular vesicles (*EV*) and uptake mechanisms. miRNAs are transcribed by RNA polymerase II from chromosomal DNA into primary RNA (*Pri-miRNA*; 1-3 kb). Pri-miRNA is processed by *Drosha* into precursor miRNA (*Pre-miRNA*). Pre-miRNA is transported to the cytoplasm and cleeved into miRNA/miRNA duplices (∼22 bp) by *Dicer*. miRNA duplex strands separate with the incorporation of the protein Argonaute (*AGO*) and RNA-induced silencing complex (*Risk*; Xu et al. 2013; Sohel 2016; Tomasetti et al. 2017). miRNA can be packed in the EV or be exported as protein-miRNA complexes (Arroyo et al. 2011; Canfran-Duque et al. 2014). Exosomes are derived from the endocytic pathway and their biogenesis requires multiprotein complexes called Endosomal Sorting Complex Required for Transport. Microvesicles are formed by the outward budding of the plasma membrane, whereas apoptotic bodies are vesicles released from cells that undergo apoptosis (Akers et al. 2013; Morrison et al. 2016). EV are taken up by recipient cells by a variety of mechanisms including endocytosis (mediated by lipid rafts, clathrin or caveolin), pinocytosis, phagocytosis and membrane fusion (Mulcahy et al. 2014). Proteins present on the EV can trigger signaling pathways in the target cells or be involved in antigen presentation (Thery et al. 2009; El Andaloussi et al. 2013). miRNA-protein complexes are also internalized by interaction with specific receptors on the recipient cell. High-density lipoprotein (*HDL*) associated with miRNA (miRNA-HDL) interacts with scavenger receptor class B type 1 (*SR-B1*; Canfran-Duque et al. 2014). miRNA-AGO-2 interacts with neuropilin-1 (*NRP1*; Prud’homme et al. 2016). *EE* Early endosome, *MVB* multivesicular body, *NPM1* nucleophosmin 1, *OMVs* outer membrane vesicles, *dsRNA* double-stranded RNA

EV are a heterogenous group of membrane-coated particles of various sizes (Fig.1) either actively or passively secreted from cells by well-established mechanisms (Thery et al. 2009; van der Pol et al. 2012; Akers et al. 2013; Ciardiello et al. 2016; Morrison et al. 2016; Table 1).

**Table 1:** Classification and characteristics (Thery et al. 2009; van der Pol et al. 2012; Akers et al. 2013; Ciardiello et al. 2016) of extracellular vesicles (*EM* electron microscopy, *ND* not determined, *TNFRI* tumour necrosis factor receptor I)

Characteristic	Exosomes	Microvesicles	Ectosomes	Membrane particles	Exosome-like vesicles	Apoptotic bodies
Size	50-100 nm	20-1000 nm	50-200 nm	50–80 to 600 nm	20-50 nm	50-500 nm and 500-4000 nm
Density in sucrose	1.13–1.19 g/ml	ND	ND	1.04–1.07 g/ml	1.1 g/ml	1.16–1.28 g/ml
EM morphology	Cup shape	Irregular shape and electron-dense	Bilamellar round structures	Round	Irregular shape	Heterogeneous
Sedimentation	100,000 g	10,000–20,000 g	160,000–200,000 g	100,000–200,000 g	175,000 g	1200 g, 10,000 g or 100,000 g
Lipid composition	Enriched in cholesterol, sphingomyelin and ceramide; contain lipid rafts; expose phosphatidylserine	Expose phosphatidylserine	Enriched in cholesterol and diacylglycerol; expose phosphatidylserine	ND	Do not contain lipid rafts	Expose phosphatidylserine
Main protein markers	Tetraspanins (CD63, CD9), Alix and TSg101	Integrins, selectins and CD40 ligand, VCAMP3, ARF6	CR1 and proteolytic enzymes; no CD63	CD133; no CD63	TNFRI	Histones, caspase3, C3b
Intracellular origin	Internal compartments (endosomes)	Plasma membrane	Plasma membrane	Plasma membrane	Internal compartments	Plasma membrane, endoplasmic reticulum

EVs have a distinct surface coat with an abundance of membrane-associated proteins, glycoproteins and lipids, whereas the EV interior (“cargo”) consists in structural and functional proteins, enzymes, lipids and peptides of various lengths. Notably, DNA (including mitochondrial DNA) and a variety of RNA species are transported within EVs. These important RNA classes have now been convincingly shown to regulate all cellular functions efficiently and include small RNAs, microRNAs and messager RNAs (Thery et al. 2009; El Andaloussi et al. 2013; Liu et al. 2015; Zaborowski et al. 2015; Morrison et al. 2016), see Fig. 1. Originally mostly ignored in electron micrographs or described as “cell-derived dust” from megakaryocytes (Wolf 1967), their original role was supposed to be passive cellular waste management. However, intriguing findings of active and orderly secretion mechanisms have emerged during the last few years. Interestingly, a wealth of information is now available that confirms that the vesicles are indeed taken up by target cells via a variety of mechanisms (summarized in Mulcahy et al. 2014) revealing their key role in intercellular communication, although targeted uptake mechanisms in any particular organ systems, except for the brain, remain to be studied in detail.

Crude urine or urinary EV (uEV), with their protein, enzyme or RNA content, have been discovered over the last few years (Alter et al. 2012; Alvarez et al. 2012; Cheng et al. 2014). In this review, we summarize some recent discoveries and highlight especially the distinct value of urinary RNA for biomarker purposes (Alter et al. 2012; Alvarez and Distefano 2013; Yang et al. 2013; Argyropoulos et al. 2015). With all the present data available, especially of the surprisingly rich and variable content of uEV, urine now appears as a very promising and completely uninvasive source for new information reflecting accurately the pathophysiology of the kidney and, most likely, also of other organ systems. Despite all the enthusiasm in this rapidly growing field, we aim to pinpoint some of the caveats, misinterpretations and limitations of current approaches and to emphasize the importance of the appropriate standardization needed.

### EV classification

Since the first descriptions of EV, a variety of vesicle categories has been described. The classification was based initially on their cellular or subcellular sources, such as prostasomes, exosomes, membrane vesicles and others (see van der Pol et al. 2012, 2016; Thery et al. 2009; Wang and Sun 2014) and basically their physico-chemical properties. These include the vesicle size, density, morphology, lipid composition, protein composition, subcellular origin and light scattering (Thery et al. 2009; van der Pol et al. 2012). As seen in Table 1, most of the physico-chemical EV characteristics overlap significantly. This also makes a reliable categorization and their isolation a challenge yet to be fully overcome. Notably, in addition to the size and density of vesicles, the efficiency of isolating these vesicles depends on the shape and volume fraction of the vesicles, the viscosity of the fluid in which they lie, the temperature and presence of other confounding factors such as proteins, peptides or pigments in the fluid, the centrifugation time and the type of rotor used for centrifugation (fixed angle or swing-out; Cvjetkovic et al. 2014; Livshits et al. 2015). Based on these variables and the current lack of thoroughly standardized isolation protocols, considerable cross-contamination of vesicle types can obviously occur in any sample studied and reported. Indeed, this overlap between vesicle categories and the variety of isolation methods might have led to “published artifacts, over-interpretation and non-comparable results between laboratories” (van der Pol et al. 2016), despite continuous attempts at standardization by international organizations (Witwer et al. 2013). On the other hand, especially for biomarker identification, the current trend is leaning towards the use of methods providing the best total EV yield, which may ignore strict EV categorization. Depending on downstream uses and goals, this should be considered acceptable, in particular as most vesicle classes share, to a significant degree, the same surface and cargo contents (even if in different ratios).

As stated above, urine contains a variety of vesicles of variable sizes (Pisitkun et al. 2004; Miranda et al. 2010; van Balkom et al. 2011; Alvarez et al. 2012; Alvarez et al. 2013). Notably, however, urinary contents, including vesicles can be strongly influenced by factors such as diet (Garcia-Perez et al. 2017), exercise (Alter et al. 2012; Yang et al. 2013; Mansueto et al. 2017) and medication and by the presence of urinary pigments. As discussed below in more detail, the lack of standardization and variables in nomenclature might have led to reported artifacts and challenges of reproducibility. Furthermore, free urine contains free and active proteases and RNases, whereas within the vesicles, both proteins and RNA are protected against ubiquitous proteases and RNases (Cheng et al. 2014). Thus, only results of crude urines are comparable with each other, whereas studies utilizing the much richer contents of EV should be preferred because of their mostly unmodified contents.

Interestingly, the bulk of urinary vesicles is considered to derive from the epithelial lining of nephrons and kidney parenchyme (Turco et al. 2016) but ample evidence has been presented that urinary vesicles also originate from the circulation (Miranda et al. 2010; Ma et al. 2016; Pazourkova et al. 2016). This offers an exciting opportunity to develop easy, non-invasive and easy-to-repeat diagnostics for remote tissue-specific markers (Ma et al. 2016) present in the urine. However, whether uEVs are actively secreted through the glomerular filtration barrier or through the epithelial cell lining of nephrons is not known, although this could involve many active mechanisms (for a variety of the mechanisms proposed, see Mulcahy et al. 2014).

Evidence is increasing for uEV usefulness in the biomarker search for kidney diseases such as minimal change disease and focal segmental glomerulosclerosis (Ramezani et al. 2015), diabetic nephropathy (Barutta et al. 2013; Musante et al. 2015; Delic et al. 2016) and others (for an excellent comprehensive review of uEV findings in kidney diseases, see Erdbrugger and Le 2016).

Although proteins within uEVs have been widely recognized and reported (see, for example, www.exocarta.org, the dedicated database for vesicle contents), the recent technical advances in RNA-sequencing (Wang et al. 2009; Lee et al. 2010) highlight the usefulness especially of urinary miRNA for biomarker purposes (Van Roosbroeck et al. 2013). Interestingly, both uEV proteins and miRNAs are products that not only are secreted by all the epithelial cell types along the nephrons and lower urinary tract but are also filtered or secreted from the circulation by the kidney parenchyme.

In contrast to earlier studies also utilizing urinary sediment cells for miRNA isolation, the protocols presently call for them being discarded as these most likely represent contents from apoptotic cells. The information available concerning specific downstream target effects of uEV miRNAs remains limited (however, see Bellingham et al. 2012; Alvarez-Erviti et al. 2011).

Interestingly, evidence is accumulating of yet another potential source of urinary RNA, namely the resident bacteria (Table 2). Accordingly, inherent but well-constrained bacterial colonization can be found in the urinary bladder (Brubaker and Wolfe 2015). For anatomical reasons, the female urinary bladder and urine are more prone to urinary tract infections (UTI). Whether this represents an escape of normal bacterial flora from the host or the entry of more pathogenic urinary pathogens, such as the uropathogenic fimbriated strains of *Escherichia coli* (Korhonen et al. 1988), remains unknown. Interestingly, the most common bacterial strains in urinary bladder associated with UTI (both Gram negative and Gram positives) have been well-established, whereas recent studies by using RNA-Seq of the 16S RNA isolated from urine have shown a wide variety of additional bacterial strains in urine (Valadi et al. 2007; Brubaker and Wolfe 2015; Koeppen et al. 2016; Tataruch-Weinert et al. 2016). At the same time, bacteria have been shown to actively use their outer membrane vesicles (OMVs) for packaging and sending their genetic material for interaction with the immediate environment. The OMV contents also include abundant small RNA species (Wang et al. 2012; Koeppen et al. 2016), which mediate interactions with host cells and tissues. Thus, urine unavoidably also contains vesicles from rich bacterial sources, with their respective RNA contents. Although little is still known of the exact roles of OMVs, they are clearly involved in the constant interplay with the host defense system and in maintaining the barrier function to prevent ascending infections.

**Table 2:** Characteristics of bacterial extracellular vesicles (*ND* not determined, *EM* electron microscopy)

Characteristics	Bacterial extracellular vesicles	
	Gram-negative	Gram-positive
Size	20-300 nm	20-100 nm
Density in sucrose	1.20–1.22 g/ml	ND
EM morphology	Round	Round
Sedimentation	150,000 *g*	150,000 *g*
Lipid composition^a^	Phosphatidylglycerol, phosphatidylethanolamine (*E. coli*)	Palmitic acid, myristic acid (*B. anthracis* and *S. pneumoniae*)
Main protein markers	Outer membrane proteins, virulence factors	Bacterial adhesion and invasion proteins, host cell modulation proteins
Intracellular origin	Bacterial outer membrane	Cell membrane
References	Lee et al. 2007; Bai et al. 2014; Kim et al. 2015; Watanabe 2016	Gurung et al. 2011; Brown et al. 2015; Kim et al. 2015

^a^Variability between strains, species

For practical purposes in uEV studies, the OMVs and their protein and RNA content are of special importance. Several questions arise. How can excessive bacterial products in the analyses and respective artifacts in the reported proteomes and RNA be avoided (Shmaryahu et al. 2014; Cheung et al. 2016)? Should samples from male and female subjects be differently interpreted as male samples present with less abundant bacteria and their OMV products? Carefulness at all steps in sample collection, storage, analysis and data interpretation is mandatory and, notably, calls for rigorous technical controls to pinpoint excessive bacterial RNA and protein products. This, in turn, may require a much larger volume of urine samples to allow all the necessary orthogonal controls (Tataruch-Weinert et al. 2016).

The capacity of bacteria to exchange functional genetic information between host cells is a widely unexplored area that has profound repercussions in our understanding of clinical UTI and beyond. The presence of bacterial proteins, metabolites and RNA products may generate misleading data and this possibility should always be critically considered in studies with uEV analytics.

Isolation methods of uEV have been extensively reviewed (Musante et al. 2014b; Wang and Sun 2014; Gamez-Valero et al. 2015). The most widely used methods are still the differential (ultra)centrifugation–based methods, often with modifications (see Table 3; Gardiner et al. 2016).

**Table 3:** Advantages and disadvantages of isolation methods for urinary extracellular vesicles (*DC* differential centrifugation, *CHAPS* 3-((3-cholamidopropyl) dimethylammonio)-1-propanesulfonate, *DTT* dithiothreitol, *SEC* size exclusion chromatography, *THP* Tamm-Horsfall glycoprotein, *uEV* urinary extracellular vesicles)

Method	Advantages	Disadvantages	
DC	Vesicle enrichment as a pellet	No standard conditions for: 1. number of centrifugations; 2. relative centrifugation force; 3. time; 4. rotor type; 5. sample volume; 6. temperature during centrifugation; 7. presence/absence of protease inhibitors	Not applicable for large volume of samples, not suitable for samples from large cohort of patients. Relatively expensive because of devise setup/reagent price
DC + CHAPS treatment	Protein activity prevention		
DC + DTT treatment	Removal of, for example, THP excess in sample	Not suitable for protein activity assessment designated samples	
DC + SEC			
Nano-membrane filtration	Removal of cell debris and urinary casts	Differences in removal of larger (>0.22 μm or more) vesicles without assessment of their importance for biomarkers screening; major loss of uEVs on the filter	
DC + microfiltration			
DC + nanofiltration			
DC + ultrafiltration			
DC + sucrose gradient	Vesicle separation according to density	Highly time consuming	
Ultrafiltration + DC			
Exoquick	No need for ultracentrifugation step	Overtly expensive when applied to large volumes	
Total exosome isolation reagent			
Hydrostatic filtration dialysis (HFD)	Inexpensive, quick, versatile. Applicable to large sample volumes and large sample numbers; no need for special machinery or highly trained personnel		

To isolate urinary EVs, a minimum of two steps are usually used, including low-speed centrifugation to pellet any cellular debris in the urine for discard.

Variations and combinations of other methods as “add-ons” to differential centrifugation have been introduced to overcome the observed limitations, including the aggregation and unnecessary loss of valuable pellets still containing EVs during the process. Notably, a number of practical parameters for the ultracentrifugation-based methods should always be carefully considered and details including relative centrifugal force, centrifugation time, temperature and the rotor type used should be recorded since variation in these parameters will lead to substantial quantitative and qualitative differences in the final EV yield (Thery et al. 2009). A detailed discussion of caveats in many of the currently used protocols can be found in recent critical reviews (Gardiner et al. 2016; van der Pol et al. 2016).

Reducing agents including dithiothreitol (DTT) or CHAPS (3-((3-cholamidopropyl) dimethylammonio)-1-propanesulfonate) are used to prevent excessive protein complexing during EV isolation, especially in order to regulate polymerization of Tamm-Horsfall glycoprotein (THP; Fernandez-Llama et al. 2010; Musante et al. 2012), the most abundant normal urinary protein distorting many downstream analyses. Notably, an intact urinary THP meshwork efficiently entraps vesicles of all sizes at all steps of EV harvesting, causes clogging and a seriously reduced isolation capacity of in-filtration-based methods and may result in a loss of up to 30 % of the final uEV yield (Fernandez-Llama et al. 2010). Notably, however, the use of reducing agents may also release miRNA bound to protective circulating proteins or from the surface of uEVs (Wachalska et al. 2016).

The current golden standard, namely serial (ultra)centrifugation, has been shown to easily miss up to 20–30 % of vesicles in the pre-purification steps (Musante et al. 2014a). Furthermore, recent results demonstrate that the final pellet after the ultracentrifugation steps for vesicle isolation may have missed yet another 20–30 % of vesicles (Musante et al. 2017). These alarming facts should be carefully taken into consideration in order to include EV isolation methods that have been selected and modified and published results that have been critically evaluated.

The most commonly used current methods and their combinations for EV isolation from urine are listed in Table 3. Notably, many of these methods can easily clog because of protein aggregates or the capacity of the method might be limited. For practical isolation purposes and for the amounts necessary especially for uEVs, large sample volumes are a definite benefit as this allows the inclusion of the necessary quality control of the samples. An additional source of artifacts, namely variations in urinary electrolyte concentrations, e.g., resulting from hydration status because of exercise (Maughan 1991), may likewise change the sample conditions and distort EV yield. Furthermore, urine is abundant in pigments, a recognized major source of artifacts in all downstream analyses. An interesting approach might be to use fluorescence-activated cell sorting (FACS) for direct vesicle isolation into various categories, although the resolution of FACS is presently limited mostly to vesicle size above 100 nm and thus misses a major part of of vesicles, especially exosomes.

Musante et al. (2014a) developed an alternative isolation method for the comprehensive catching of urinary vesicles. This method is called hydrostatic filtration dialysis (HFD) and avoids the recognized limitations of most EV isolation methods. It uses simple and quick low-speed centrifugation (2000 *g*) to remove, for example, bacteria, cellular debris and excessive polymers of THP before filter-dialysing the sample (especially urine).

In HFD, the hydrostatic pressure of the sample pushes it through the dialysis membrane tubing. For recovery of urinary proteins and miRNA, the HFD method is clearly superior to the ultracentrifugation-based methods and provides superior quality of protein and RNA yield (Musante et al. 2014a; Tataruch-Weinert et al. 2016).

HFD is versatile, simple and inexpensive and can be used for a variety of samples including urine, plasma, cell culture medium and saliva (Musante et al. 2014a). It is based on sample dialysis in a tube with a defined-cutoff pore-size membrane (Musante et al. 2012). This membrane allows the passage of small solutes and peptides, effectively standardizing the solute environment, while retaining the EVs because of their larger size. HFD easily provides normalization and collection of the yield and washes away electrolytes while efficiently retaining the vesicles. Notably, the major contaminant in all urinary downstream analyses, namely urinary pigments, are also washed though the membrane and thus are avoided. The capacity of this method is adequate, with up to 1000 ml sample volumes being easily handled. Furthermore, this method requires little or no previous knowledge or training, is extremely cost-efficient and can readily process a number of samples in parallel during a workday (Musante et al. 2014a). The HFD method is not suitable for distinguishing between defined vesicle populations, which, for most diagnostic, prognostic or biomarker searches is, however, irrelevant.

Antibodies and synthetic peptides with affinity for EV membrane proteins have been used for EV isolation (Ghosh et al. 2014; Wang and Sun 2014). Although these methods may preferentially yield exosomes or other EV types with distinct surface antigens as required, additional EV subpopulations sharing the same membrane proteins may be isolated. Moreover, their capacity for EV catching is limited making them more suitable for smaller sample volumes.

Specific kits to precipitate exosomes have been developed, e.g., ExoSpin Exosome Purification Kit (Cell Guidance Systems, USA), Invitrogen Total Exosome Isolation Kit (Life Technologies, USA) and others. These products have been tested with solutions of liposomes and exosomes and have shown not only a robust isolation capacity for EV but also the co-isolation of molecules with similar physical properties. Thus, other additional purification methods may need to be used (Lane et al. 2015).

### RNA species in vesicles

Intracellular RNA species are involved in the translation of DNA information to proteins at ribosomes and in the general regulation of RNA translation (see Grosshans and Filipowicz 2008, Xu et al. 2013). MiRNA, with the size of 20-25 nt, has a distinct role in gene regulation (Xu et al. 2013). Its formation is regulated by a specific intracellular pathway distinguishing it from other small RNA species present both in pro- and eukaryotic cells (Xu et al. 2013; see Fig. 1).

Interestingly, all RNA classes can also be found extracellularly, in all bodily fluids, including serum and plasma, saliva, tear fluid and urine (Wang et al. 2012; Patton et al. 2015; Yanez-Mo et al. 2015). The way in which the cellular RNA secretion itself is regulated and the relative proportions of the various small RNA species secreted are not exactly known. The intracellular RNA profile may differ from that of the secreted profile, suggesting the possibility of the overflow being secreted. Packaged into EV but also free, RNA appears as a unique and archaic method of cell-to-cell communication found throughout living cells (Brown et al. 2015; Yanez-Mo et al. 2015). Interestingly, the RNA secretome appears to reflect accurately the physiological state of the cell of origin and is highly valuable and accurate for the mapping of any personalized functions.

Although free extracellular RNA was identified years ago, the extracellular milieu consists in complex proteases and ubiquitous RNases actively splicing any target secreted from the cells (Sorrentino 1998; DeClerck et al. 2004; Ricard-Blum and Vallet 2016). The splicing process produces the extracellular fluid proteome (peptidome) and RNome. What is even more exciting is that cells ranging from archaic bacteria to the most highly sophisticated cell types have developed methods to protect these valuable entities from degradation by packaging them into vesicles and, indeed, extracellular RNAs appear to be universally associated instead with carrier vehicles (Patton et al. 2015), probably because of the rapid degradation of unprotected RNAs in biofluids.

Exosomes appear as a dominant pathway and vehicle for RNA secretion from cells (Crescitelli et al. 2013; Lunavat et al. 2015; Willms et al. 2016), although other vesicle types also contain RNA. This fact reduces the value of any method designed strictly for the purification of a distinct EV class. Moreover, these methods always end up in losing up in 40 % of EV because of losses in the process up until the last separation steps (Musante et al. 2017). Furthermore, the membrane layer of all vesicle types has been shown to contain a distinct surface coat that may be quite different from the vesicle interior, i.e., the “vesicle cargo” (Liu et al. 2015). This is interesting, as many lines of evidence now suggest different roles for these regions: the surface proteins and even EV surface RNA may serve as an “address code” for targeting (for references, see Mulcahy et al. 2014). As the vesicle attaches and fuses with the target cell membrane, its contents are released for intracellular interaction. This appears as a finely tuned and highly sophisticated system for target cell regulation in processes such as immunological defense, cancer spread and the modulation of the metabolic state of cells in a specific manner (El Andaloussi et al. 2013; Yanez-Mo et al. 2015; Zaborowski et al. 2015).

The gut, skin, urine and mucous membrane microbiomes appear to have developed methods to efficiently interfere with the host defense systems by releasing outer membrane vesicles (OMVs), with distinct RNA contents and constant interaction with the host defense system. As a result, a strong, although less-well understood barrier function is achieved.

## Future aspects

The number of studies on EV, especially exosomes, has been sky-rocketing during the last few years. This is because their extraordinary potential is not limited to the dissection of disease mechanisms to identify new druggable targets but can also provide early biomarkers of unforeseen accuracy. EV, which are also abundantly present in the urine, are an unparalleled resource for individualized molecular fingerprints reflecting cellular pathophysiology at their site of origin upstream in the kidney and beyond (Miranda et al. 2010; Alvarez and Distefano 2013). Thus, EV are derived not only from all cell types along the nephron and kidney parenchyme (Salih et al. 2014) but also from the circulation and normal bacterial colonization in the urinary bladder.

The potential of EV, especially for early diagnostics and as novel biomarkers for kidney diseases, is exciting. With full systems biology integration, combining their proteome, RNome and metabolomics information, EV now show great promise in dissecting these thus far less-well understood disease mechanisms in an individualized way (Erdbrugger and Le 2016; van der Pol et al. 2016). The field still lacks comprehensive standardization, starting from the nomenclature and methods used for efficient EV harvesting and extending up to their critical downstream analysis. Misleading or false results in the field may lead to wrong conclusions (van der Pol et al. 2016). However, the challenges have been recognized and respective international organizations are committed to continuous standardization (Witwer et al. 2013). Novel, inexpensive and critically scrutinized methods are being developed in order to avoid the gaps of the earlier ones and to match expectations, for example, for personalized markers.

Whereas the physical properties and protein and RNA content of the various EV types significantly overlap, a careful selection of methods available should provide a rational basis for an appropriate selection of each type of application (Musante et al. 2014b; Rupert et al. 2017).

Notably, methods for single vesicle detection are developing rapidly, including those utilizing microfluidics and nano-sized beads. These will be highly useful, especially for linking the different vesicle types to specific contents and, thus, for revealing details of their functions. The respective compact systems and techniques including micro-nuclear magnetic resonanace (Shao et al. 2012), magneto-electrochemical sensor systems (Jeong et al. 2016) and nanoplasmonic chips (Im et al. 2014) are expected to develop into practical and rapid point-of-care detection for diagnostics, especially for uEV-based protein miRNA biomarkers (Shao et al. 2015). Flow cytometry is currently developing as an exciting extension for EV isolation directly in suspension and provides a means for the efficient identification of EV based on their type-specific epitopes and sequestration into subpopulations. However, currently, the size-range of flow cytometric detection starts at 200 nm going upwards, although a detection limit down to 100 nm has been achieved (Pospichalova et al. 2015; Rupert et al. 2017). Thus, FACS detection, especially of exosomes, is currently beyond the detection limit (Shao et al. 2015).

The massively parallel Next-Generation Sequencing as applied also to uEV analytics is a welcome extension to the repertoire of the novel robust and non-biased methods for urinary analytics (Renkema et al. 2014; Khurana et al. 2017). Carefully planned studies, meticulous controls and critical evaluation of results will provide an unforeseen accuracy to mechanisms of health and disease beyond the kidney parenchyme and urinary tract. The obvious caveat of genetic material as a “contaminant” from bacterial OMVs should be noted. Moreover, novel mechanisms to distinguish bacterial from “non-bacterial” cystitis, an important clinical problem, are expected to be discovered with emerging new therapeutic options.

An exciting extension to utilizing EV biology relates to bioengineered nanoparticles. These can serve as EV mimics and can be released into the circulation with a defined target cell “address code”. Upon attachment, the engineered protein, regulatory RNA or selected medication contents can be released as previously exemplified by the delivery of targeted chemotherapeutics (Jang et al. 2013). These treatments will have a huge influence, including on kidney diseases, with next-generation targeted therapies fully utilizing the obtained knowledge of EV biology and their bioengineering capacities.
